# Characteristics of Porous Aluminium Materials Produced by Pressing Sodium Chloride into Their Melts

**DOI:** 10.3390/ma14174809

**Published:** 2021-08-25

**Authors:** Iva Nová, Karel Fraňa, Pavel Solfronk, Jiří Sobotka, David Koreček, Martin Švec

**Affiliations:** 1 Department of Technology, Faculty of Mechanical Engineering, Technical University of Liberec, Studentská 1402/2, 461 17 Liberec, Czech Republic; iva.nova@tul.cz (I.N.); pavel.solfronk@tul.cz (P.S.); jiri.sobotka@tul.cz (J.S.); david.korecek@tul.cz (D.K.); martin.svec@tul.cz (M.Š.); 2 Department of Power Engineering Equipment, Faculty of Mechanical Engineering, Technical University of Liberec, Studentská 1402/2, 461 17 Liberec, Czech Republic

**Keywords:** porous aluminium materials, sodium chloride, melt, AlSi12, pressing, mechanical properties

## Abstract

The paper deals with research related to the production of metal cellular aluminium systems, in which production is based on the application of sodium chloride particles. In this paper, the properties of porous aluminium materials that were produced by an unconventional method—by pressing salt particles into the melt of aluminium alloy—are described. The new methodology was developed and verified for the production of these materials. The main feature of this methodology is a hydraulic forming press and a simple-shaped foundry mould. For these purposes, four different groups of sodium chloride particle sizes (1 to 3, 3 to 5, 5 to 7 and 8 to 10 mm) were applied. The preferred aluminium foundry alloy (AlSi12) was used to produce the porous aluminium samples. Based upon this developed methodology, samples of porous aluminium materials were produced and analysed. Their weight and volume were monitored, their density and relative density were calculated, and their porosity was determined. In addition, the porosity of samples and continuity of their air cells were monitored as well. An industrial computed tomograph and a scanning electron microscope were applied for these purposes.

## 1. Introduction

Within the metallurgical industry, there is a constant focus on the production of cellular (lightweight) metal materials. The basic characteristics of these materials were already presented by Ashby [1] in 1983. Other publications about the production and properties of metallic cellular systems include, e.g., the publications of Banhart [2,3], which state that there are four basic types of production of cellular metals (“foaming metals”): direct melt foaming, controlled solidification of the melt in a supersaturated gas atmosphere, powder metallurgy and foundry methods. At the same time, there are efforts to make the production of these materials more environmentally friendly. It is clear from the research published in [4] that there is currently such an interest in the production of cellular metals and the evaluation of their properties and that there are already about 400 researchers who publish facts about these materials.

Generally, foundry methods related to cellular metals can be divided into three groups (lost body casting, filler material casting [3] and replication processes [5,6,7]). Metallic cellular systems (porous metals and foaming metals) are lightweight metallic materials, which contain intentionally formed pores in their structure. In this paper, we focus on porous aluminium materials—sometimes also described as structures with closed or opened porosity.

The production of metallic cellular systems based on the use of sodium chloride particles is very advantageous. Such kinds of production mostly use a replication process. The first report on the replication process was published in the early 1960s and, since then, the research at the Swiss Federal Institute of Technology in Lausanne developed across various stages. Luna [7] reported the entire replication process in detail. Such technology is based on the use of vacuums and represents the standard method for production of open-cell metal foams at the Department of Material Science and Engineering at the University of Sheffield. Further research was carried out at this department and two other new methods were derived there—termed as gas and mechanical infiltration.

These porous materials have many free voids (pores or cavities) in their structure. The size of the pores varies, but they are most often in the scale of millimetres. In engineering, a homogeneous material is quite often preferred. However, in this case, the reverse effect is applied, i.e., that pores have a certain size in the same structure [8,9,10,11,12]. Foaming metals with open cells are also sometimes referred to as porous metallic materials. These materials contain so-called open cells with a tailored porous morphology that is produced and is based upon such particles as sodium chloride. These NaCl particles are filled with a melt of metal alloys such as, e.g., aluminium alloys. The solidification of these melts produces a heterogeneous system consisting of metal and sodium chloride particles.

As stated by [13,14], the nature of porous material depends on the pore size, as determined by IUPAC (International Union of Pure and Applied Chemistry). The following three classifications are mentioned: microporous, mesoporous and macroporous materials—their definitions are given in publications [13,14]. This porous material is commonly used, e.g., for the production of filters, fuel cell anodes or stationary parts for chromatography. In addition to that, they can be also used for the production of microfluidic chips and parts for heat transfer [14].

Various technologies are used in the production of porous materials [15,16,17,18,19]. There are also known works that use sand particles bonded, e.g., with furan resin, or ceramic particles bonded with gypsum instead of sodium chloride. Hussain [17] deal with the produstion of porous aluminium samples based on the sintering powder and sodium chloride particles. It was concluded that the preferred amount for this purpose is 60 wt% NaCl. The porous aluminium samples that were produced revealed the highest energy absorption. Generally, in all manufacturing methods, the mechanism by which the melt penetrates between the particles of substance that defines the voids in the porous material is crucial. The most common method is infiltration of the metal melt, also known as the replication process [18]. The authors of [20] applied sodium chloride to produce lead-based porous materials with some amount of antimony (25 wt%) for the purpose of producing grids for electric batteries. The authors refer to this method used to produce porous materials as the Excess Salt Replication Method (ESR method). Methods for preparation of the aluminium alloys for experimental and practical purposes are discussed in the works of [21,22].

The authors of [23] based the production of porous materials on powder metallurgy by using spherical granulated carbamide as well as sodium chloride. The authors of [24] also dealt with the production of porous aluminium materials. In the production of these materials, they used a foaming agent (CaCO_3_) that was added to the prepared aluminium melt. Furthermore, a fusion method was used in which they also added NaCl particles into the melt. Such combination of these two substances (CaCO_3_ and NaCl) added to the melt slightly contributed to its foaming process. The fusion method that was used did not provide the expected results for its application in terms of producing porous aluminium materials. The authors of [25] used sucrose for the production of porous aluminium materials. The researchers of [26] studied the mechanical properties of aluminium foams. In [27], the production of porous aluminium materials by means of pressing an aluminium alloy melt among the grains of sodium chloride was investigated. In this way, it was possible to produce circular samples with quite small height (60 mm × 10 mm). Publication [28] presents the results of sodium chloride thermal analysis. Based upon these results, sodium chloride was used for the production of porous aluminium samples. The first experiments provided preliminary information demonstrating possibilities in the production of aluminum porous materials. These results of the first findings were already published in [27]. In other experiments, we applied the idea of pressing. Firstly, we pressed the melt between the sodium chloride grains. This production technique showed that a mould needs to use comparable amounts of sodium chloride and aluminum alloy particles. Gradually, we developed a methodology for pressing previously prepared sodium chloride particles into the melt. On the obtained samples, we tentatively tested the strength, which was the main subject referred to in [29]. This method of sample production was further improved and other sodium chloride particle sizes were used. Simultaneously, the interconnection of pores of manufactured porous systems was investigated in detail using the METROTOM 1500 CT ZEISS and discussed in this paper.

The research published in [30] compared two production methods of metal foams based on powder processing, the Powder Compact Melting Technique (PCMT) and the Sintering and Dissolution Process (SDP), respectively. The PCMT method is based on the melting of powders. The SDP method is based on Al powder and NaCl particles. These components are processed successively by mixing, compaction, sintering and finally dissolving the NaCl particles. Static pressure tests were performed on the aluminum foams produced using both methods. PCMT foams showed higher mechanical properties in pressure tests compared to SDP foams. The results confirmed that PCMT foams are ideal for structural applications in which energy absorption is the main task. SDP foams have an interconnected porosity and are useful in absorbing noise and vibration. The examples of produced porous aluminium materials are shown in [31].

In the current paper, the melted form of the material was used, and the particles of the NaCl were used in pressing. The NaCl particles were treated by heat, as is explained in detail later. 

## 2. Materials and Methods

The experimental production of porous Aluminium materials, following our first publication [27] and [29] further experiments, was carried out to obtain porous aluminium materials based on the knowledge of sodium chloride behaviour at high temperatures (the melting temperature of sodium chloride is about 801 °C). This temperature was high enough to perform experiments with aluminium alloys and sodium chloride. For the purposes of the research, two samples of sodium chloride were used—sample No. I. (from which medium-sized particles of 2 and 4 mm were separated) and sample No. II. (from which medium-sized particles of 6 and 9 mm were separated). Chemical analysis of both sodium chloride samples was performed by means of the X-ray fluorescence method on the XRF Bruker S8 Tiger device (Billerica, MA, USA). The chemical composition of both samples is given in Table 1.

**Figure 1:**
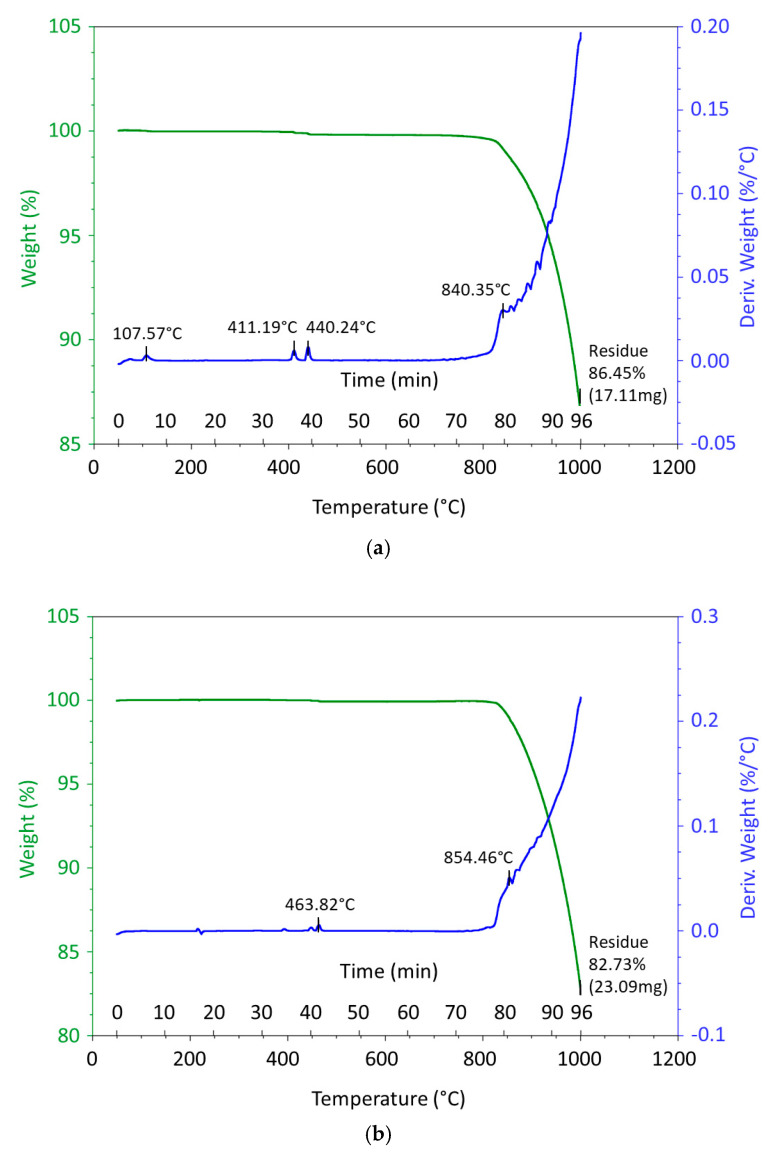
Results of thermal analysis and differential thermal analysis of sample Na Cl I. (**a**) and NaCl II. (**b**).

Thermal analysis (TG/DTA—thermogravimentry/Differential Thermal Analysis) of samples No. I. and No. II. was performed on a Discovery Series device (TA Instruments, company Bruger, New Castle, DE, USA). Both samples were heated up at a heating rate 10 °C·min^−1^ in flowing air. The results of the thermal analysis and differential thermal analysis of samples No. I. and No. II. NaCl are shown in Figure 1.

On the basis of the above theoretical overview, it is clear that successful production of porous aluminium materials using sodium chloride must be based, for example, on the use of a vacuum system and possibly an inert gas (argon). The production of porous aluminium materials using particles of sodium chloride (salt cores) or the space holder particles (SHP) method is most often based on a melt infiltration process. At our department (Department of Engineering Technology, Faculty of Mechanical Engineering, Technical University of Liberec), the production of porous metallic materials was also solved as part of a grant project. The production of porous aluminium material by pressing of the melt between sodium chloride particles was described in [27], and an evaluation the properties of the material was thus produced. In this publication, the results concerning the production of porous aluminium material by pressing sodium chloride particles into this melt are presented. The main aim was to prove that, by using production techniques based on pressing, it is possible to produce porous type materials with the specific material properties defined by, e.g., density, etc. These structure samples were analysed on the computer 3D tomograph (CT METROTOM, model 1500, company ZEISS, Jena, Germany) and it was clearly demonstrated that the resulting materials structures are in the form of open cells. 

For the production of aluminium porous materials, an unconventional technology, based on pressing the sodium chloride into the aluminium alloy melt in a mould, was designed and tested. For this purpose, an AlSi12 aluminium alloy (EN AC-44300, melting temperature approx. 577 °C), with a density of 2650 kg·m^−3^, was used. The chemical composition of the tested aluminium alloy used was identified using a Bruker Q4 Tasman optical emission spectrometer, see Table 2.

**Figure 2:**
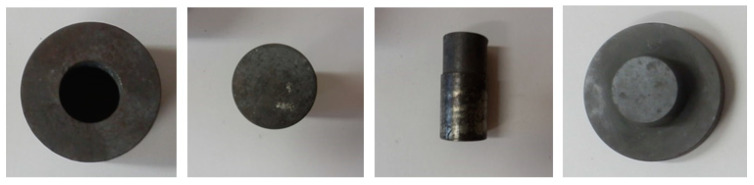
A view of the parts of a steel foundry mould.

For pressing of the sodium chloride into the melt of aluminium alloy AlSi12, a steel foundry mould, produced according to the Czech norms ČSN EN 1.2343, was used. The mould cavity was shaped as a conical cone and consisted of four parts, as can be seen in Figure 2. 

The cylindrical part had the following geometry—90 mm outer diameter, 110 mm height, 40 mm holes for punching, 47 mm holes for ejecting the porous part from the mould. The dimensions of punch were 38 mm × 80 mm. In addition, there was also a spacer with a truncated cone (100 mm × 45 mm) and conical cone with dimensions of D_1_ = 51 mm, D_2_ = 46 mm and a height of 25 mm.

**Figure 3:**
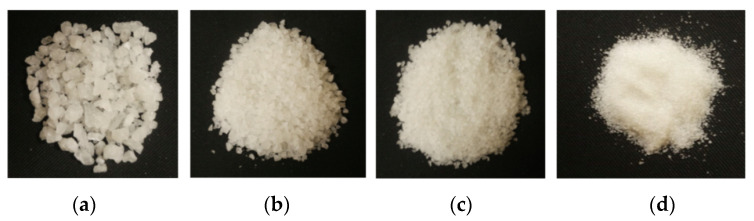
Sodium chloride particle sizes for the production of aluminium porous materials (**a**): 8 to 10 mm; (**b**): 5 to 7 mm; (**c**): 3 to 5 mm; (**d**): 1 to 3 mm.

For the production of the aluminium cellular system (more precisely, porous aluminium alloy) a combination of the foundry method with the utilization of filler material was used. This material was sodium chloride. Four particle size groups were used for the experiments: 1 to 3 mm; 3 to 5 mm; 5 to 7 mm and 8 to 10 mm (see Figure 3). In terms of physical properties, the melting temperature of sodium chloride Tm = 801 °C and enthalpy of melting Hm = 488,000 J·kg^−1^ were important for the production of the porous material.

### Calculation the Amount of Salt and Alloy for Production of Aluminium Alloy Porous Samples

The samples were made in the mould with truncated cone cavity. Calculation of the truncated cone cavity volume was performed according to the general formula:

(1) $$V=\frac{1}{12}\;\pi \times v\times \left(D^{2}+D\times d+d^{2}\right)$$

Values for the calculation were as follows: *D* = 47 mm, *d* = 40 mm and *v* = 40 mm. By substituting the values into Equation (1), the mould cavity volume (*V*) could be calculated (*V* = 5.689 × 10^−3^ m^3^). During the production of the aluminium porous system, it was assumed that 50% of the mould volume *V* would be filled with aluminium alloy (AlSi12) and 50% of the mould volume would be filled with sodium chloride NaCl. This ratio between amounts of salt and melt was prefered from the point of the view of the experiments, as it was visually easier and more practical to control the amounts of both components in the experiments and also in real production. It can be calculated that the volume of the alloy and sodium chloride is:

(2) $$V_{AlSi12}=0.5\times V=2.987\times 10^{-5}\;(m^{3})$$

(3) $$V_{NaCl}=0.5\times V=2.987\times 10^{-5}\;\left(m^{3}\right)$$

Furthermore, Equation (3) was used to calculate the mass m of sodium chloride:

(4) m=ρ×V

where *m* is weight (kg); ρ is density (kg·m^−3^); *V* is volume (m^3^).

Table 3 shows the calculated weight values of AlSi12 alloy and sodium chloride. 

Table 3 shows the amount of the salt and melt used for all of the produced samples that involved the influence of the different sizes of the salt particles, respectively. The amount is expressed by volume and density. The salt component contains the specific amount of air, which depends on the shape of the salt particles. Because the particles are of non-spherical shape, air is randomly and non-uniformly distributed. Further details about this fact can be found in [31]. 

Prior to placing NaCl on the melt surface into the preheated foundry mould, the NaCl was heat treated (furnace temperature of 300 °C, time 1 h) so as to interconnect the gas cells in the porous material. The NaCl was treated by heat in order to control the humidity and to create a compact from the salt particles, leading to the walls of the resulting materials being more homogeneous. This corresponds well to the SDP method described in [30].

Aluminium alloy AlSi12 was melted in a graphite crucible in a “Classic” resistance melting furnace. The preheating temperature of the melt was 720 to 750 °C. The melt was metallurgically treated with refining salts before casting into the mould and, after the temperature was measured, the melt was poured into the mould. Before pouring the melt into the mould cavity, the mould was preheated to a working temperature and treated with a protective graphite spray (Molybkombin UMF T4 spray). After that, a measured amount of aluminium alloy AlSi12 was poured into the preheated mould and a measured amount of sodium chloride was poured in as well.

**Figure 4:**
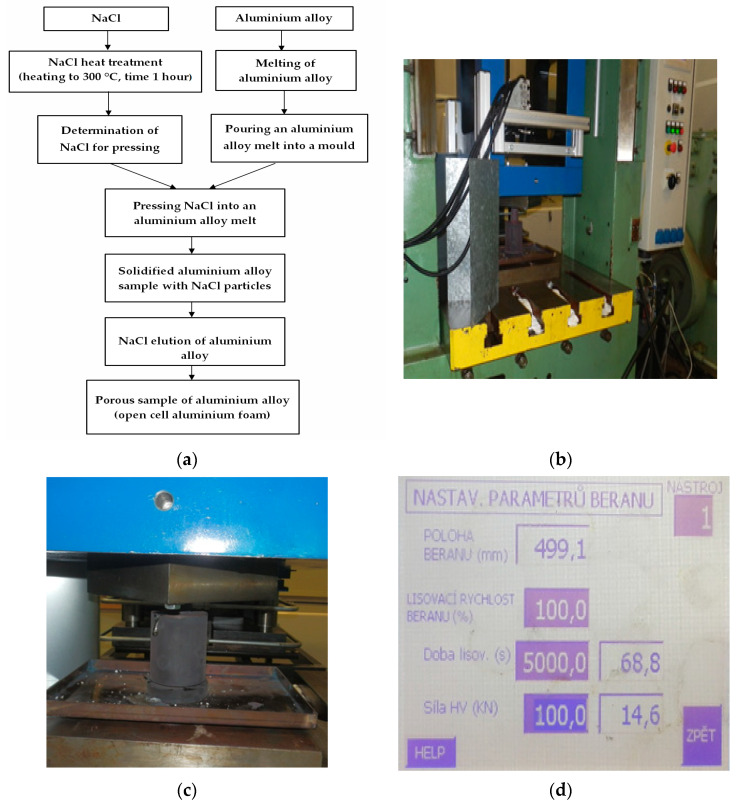
(**a**) Scheme of production porous materials. (**b**) View of the hydraulic press. (**c**) Detailed view of the foundry mould with NaCl pressing and (**d**) its monitor with the settings of the required presses values (position of the press ram: 499.1 mm; pressing speed of the ram: 100; pressing time: 5000 s; pressing force: 100 kN).

In Figure 4a, the schamatic describes this production process for the samples.

It is necessary that the hydraulic press is well adjusted for pressing (see Figure 4b). It is also very important that the pressing parameters of the machine are properly set (in this case, the ram position was 499.1 mm, the ram pressing rate was 100, the pressing time was 5000 s and the pressing force was 100 kN). For a detailed view of the hydraulic press monitor, see Figure 4d. Figure 4c shows the pressing of the melt with sodium chloride in a mould that was inserted under the punch of the hydraulic press. A minimum pressure of 80 MPa was applied on the sodium chloride in the mould.

After pressing the AlSi12 aluminium melt with the corresponding content of sodium chloride particles in the mould, solidification of this melt took place within a time period of 15 s. An aluminium alloy, with a spatial distribution of sodium chloride particles throughout the volume of the produced sample, was obtained. Thus, the produced samples were immersed in boiling distilled water, where they remained for 1 h. Such a procedure dissolved the sodium chloride particles. After the dissolution process, the produced samples were left in the dryer for 1 h to remove any remaining distilled water.

After drying for one hour, the weight of the samples was determined using the double mass weighing on the air and the water, respectively. The weight of the samples was determined using the RADWAGWPS, model 4000/C/2 electronic balance, company RADWAG, Radom, Poland with an accuracy of 0.01 g. The water solution with sodium chloride was further dried and its weight was checked, thus monitoring the removal of salt from the produced sample. 

**Figure 5:**
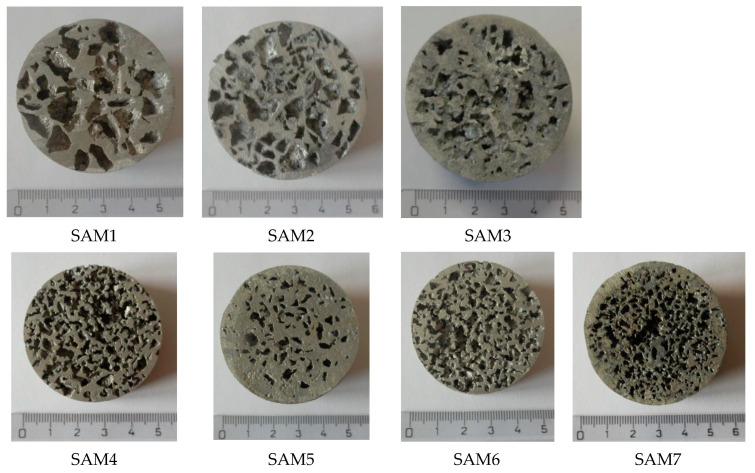
Manufactured samples of porous aluminium materials from AlSi12 alloy in the shape of a truncated cone.

**Figure 6:**
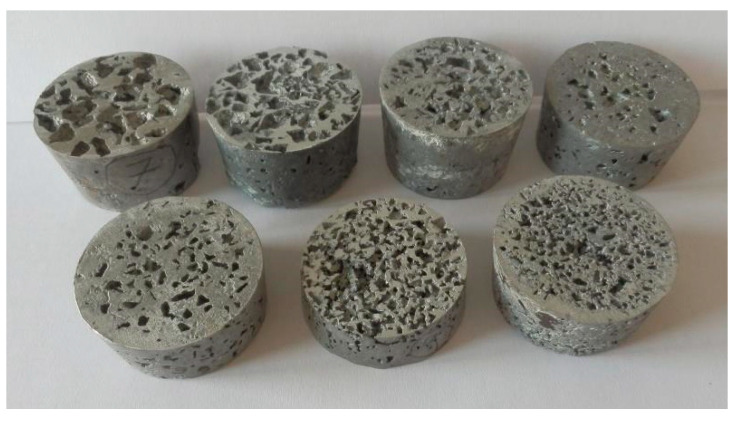
View of manufactured samples of aluminium porous material, AlSi12 alloy, in the shape of a truncated cone (the samples are not sorted by number).

In Figure 5 and Figure 6 are shown the samples of porous aluminium materials, with all four used particle sizes of sodium chloride, that were produced by pressing them into the *AlSi12* melt. The weight of the samples and their dimensions are given in Table 4. 

## 3. Results

The produced samples of aluminium porous materials were analysed. At the same time, physical values were calculated based on their material characteristics, which are shown in Table 4. For determination of their weight, the RADWAG WPS, model 4000/C/2 electronic equipment, company RADWAG, Poland was used. The essential parameter for the experiment was the volume of the sample in which the uncertainty of values could be calculated according to the approach mentioned in Appendix A. 

### 3.1. The Properties of Porous Materials

Evaluation of the porous aluminium alloy AlSi12 material properties produced by pressing NaCl into an aluminium alloy melt.

To evaluate the properties of prepared porous aluminium alloy system, a methodology was developed to determine the relevant physical-material characteristics or other relevant quantities. Such an evaluation of the produced aluminium porous materials involves the determination of the following quantities:

Determination of porous material density (ρ_Al.P._):

(5) $$\rho_{Al.P.}=\frac{m_{Al.P.}}{V_{Al.P.}}$$

where ρ*_Al.P_*_._ is density (kg·m^−3^); *m_Al.P._* is weight (kg); *V_Al.P._* is volume (m^−3^).

Determination of the porous material relative density (ρ_REL_):

(6) $$\;\;\rho_{REL}=\frac{\rho_{P.\left(AlSi12\right)}}{\rho_{B.\left(AlSi12\right).}},$$

where ρ*_REL_* is the relative density (1); ρ*_AlSi12, P._* is the density of porous aluminium alloy (kg⋅m^−3^); ρ*_B._*_(*AlSi12*)_ is the density of the base material (without porosity) (kg·m^−3^).

Determination of material porosity (*P*) is the ratio of the density difference between the base material and the porous material (porous aluminium—*Al. P.*) to the density of the base (non-porous) material (*B.M.*):

(7) $$P=\left(\frac{\rho_{B.M.}-\rho_{Al.P.}}{\rho_{B.M.\;}}\right)\cdot 100\;\left(\%\right)=\left(1-\frac{\rho_{Al.P.}}{\rho_{B.M.}}\right)\;\cdot 100\;\left(\%\right)$$

Based on Equations (5)–(7), the chosen values of the produced porous system, made of aluminium alloy AlSi12, were calculated. These values are given in Table 5 and Table 6. 

**Figure 7:**
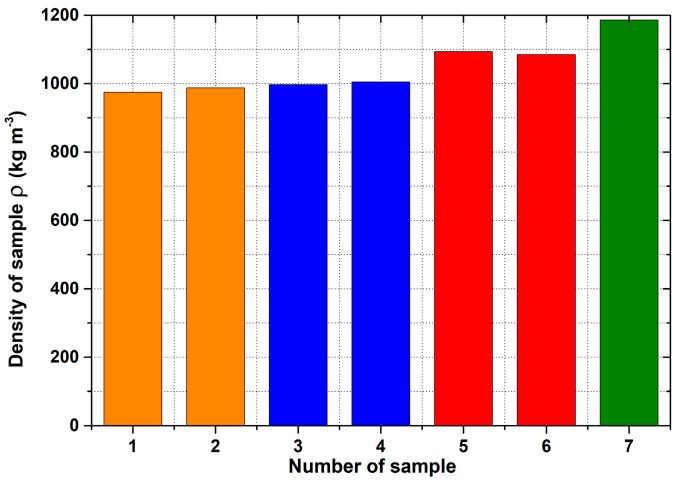
Density values of individual aluminum porous samples. Orange colour: 1—SAM1; 2—SAM2. Blue colour: 3—SAM3; 4—SAM4. Red colour: 5—SAM5; 6—SAM6. Green colour: 7—SAM7.

**Figure 8:**
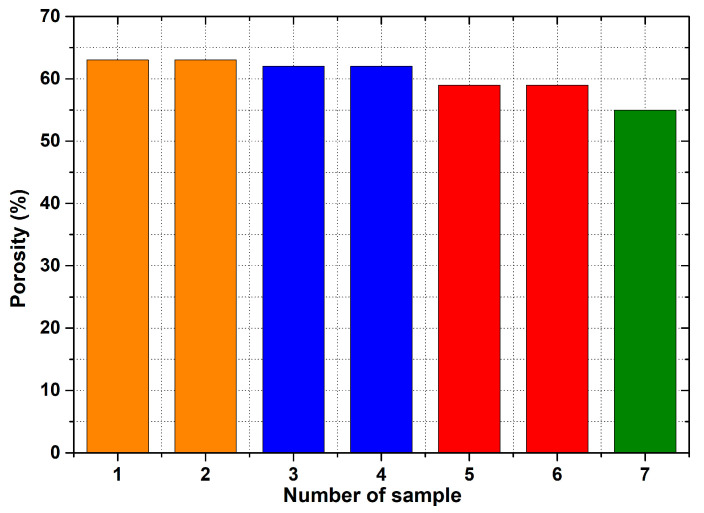
Porosity values of individual aluminum porous samples. Orange colour: 1—SAM1; 2—SAM2. Blue colour: 3—SAM3; 4—SAM4. Red colour: 5—SAM5; 6—SAM6. Green colour: 7—SAM7.

Figure 7 shows the density of the samples and Figure 8 depicts their porosity. 

### 3.2. Structure of Porous Materials

**Figure 9:**
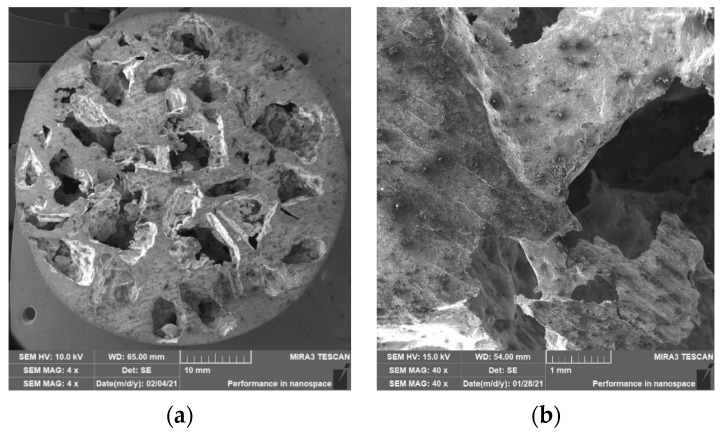
View of the structure of the porous material of AlSi12 alloy: (**a**) detail of the material; (**b**) scanning electron microscope image (Vega 3 Tescan, SEM HV 20.0 kV) of sample SAM1.

**Figure 10:**
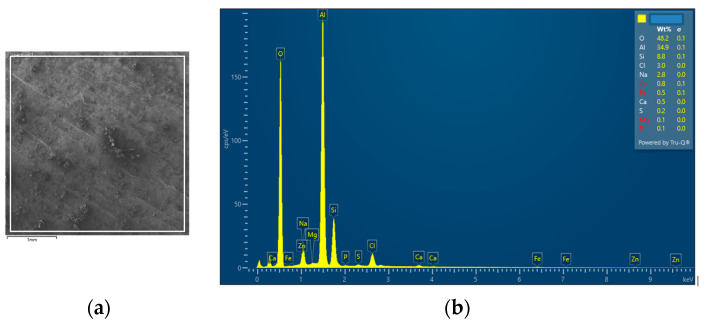
EDX analysis and its evaluation with respect to the local chemical composition: (**a**) chemical composition of the AlSi12 alloy used; (**b**) sample SAM 1.

**Figure 11:**
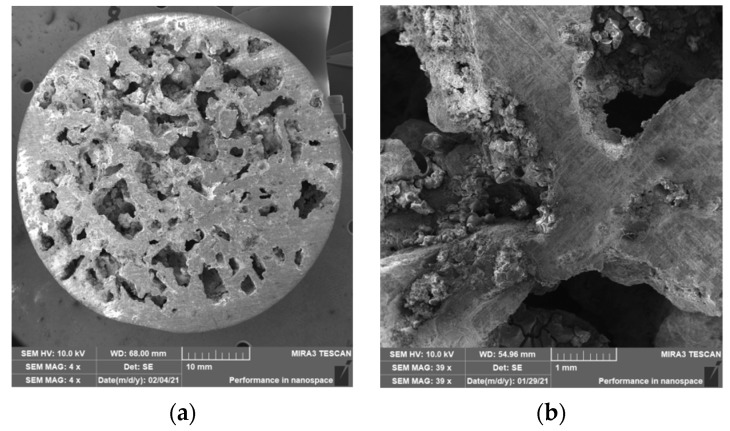
View of the structure of the porous material of AlSi12 alloy: (**a**) detail of the material; (**b**) scanning electron microscope image (Vega 3 Tescan, SEM HV 20.0 kV) of sample SAM3.

**Figure 12:**
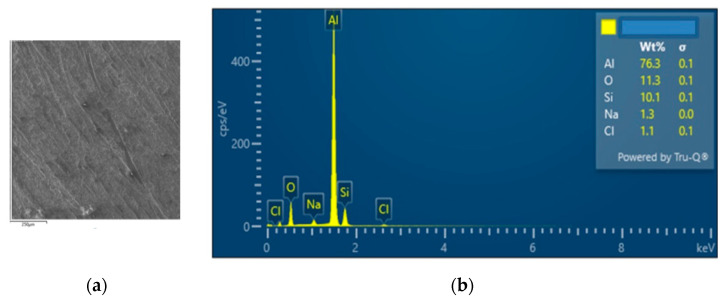
EDX analysis and its evaluation with respect to the local chemical composition: (**a**) chemical composition of the AlSi12 alloy used; (**b**) sample SAM3.

**Figure 13:**
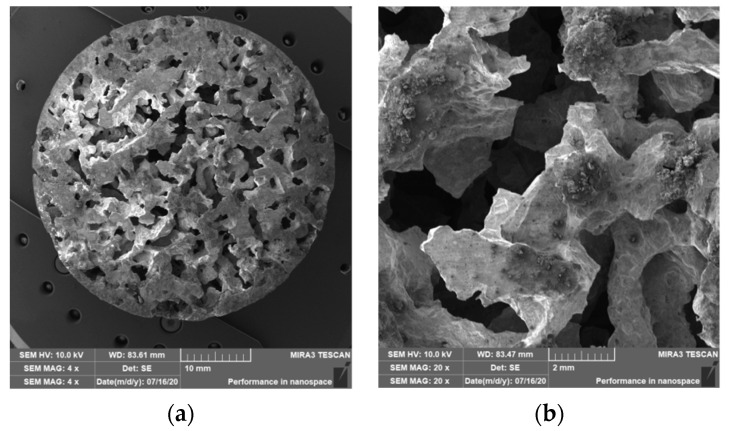
View of the structure of the porous material of AlSi12 alloy: (**a**) detail of the material; (**b**) scanning electron microscope image (Vega 3 Tescan, SEM HV 20.0 kV) of sample SAM4.

**Figure 14:**
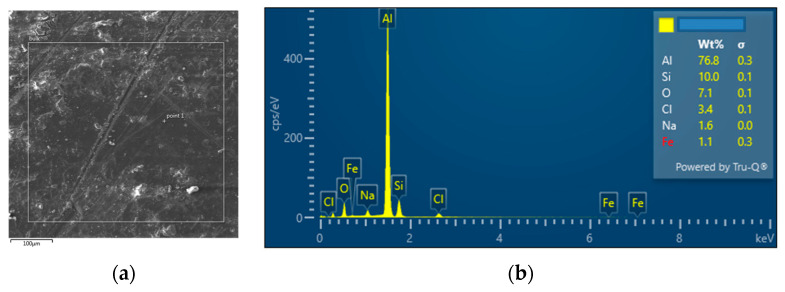
EDX analysis and its evaluation with respect to the local chemical composition: (**a**) chemical composition of the AlSi12 alloy used; (**b**) sample SAM4.

**Figure 15:**
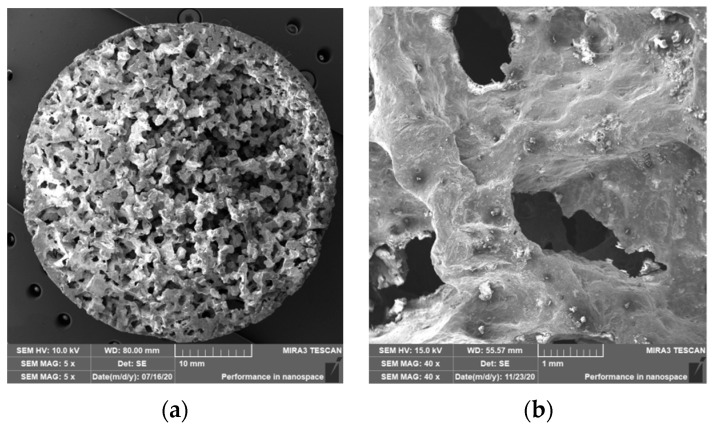
View of the structure of the porous material of AlSi12 alloy: (**a**) detail of the material; (**b**) scanning electron microscope image (Vega 3 Tescan, SEM HV 20.0 kV) of sample SAM5.

**Figure 16:**
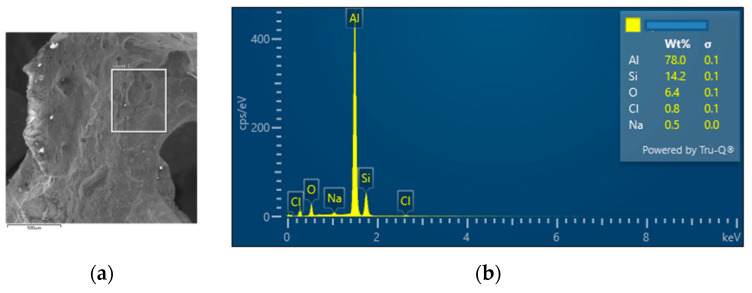
EDX analysis and its evaluation with respect to the local chemical composition: (**a**) chemical composition of the AlSi12 alloy used; (**b**) sample SAM5.

**Figure 17:**
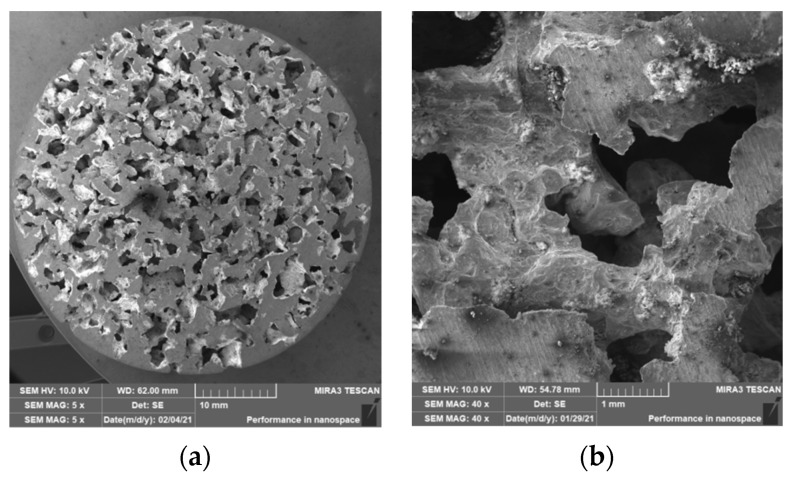
View of the structure of the porous material of AlSi12 alloy: (**a**) detail of the material; (**b**) scanning electron microscope image (Vega 3 Tescan, SEM HV 20.0 kV) of sample SAM6.

**Figure 18:**
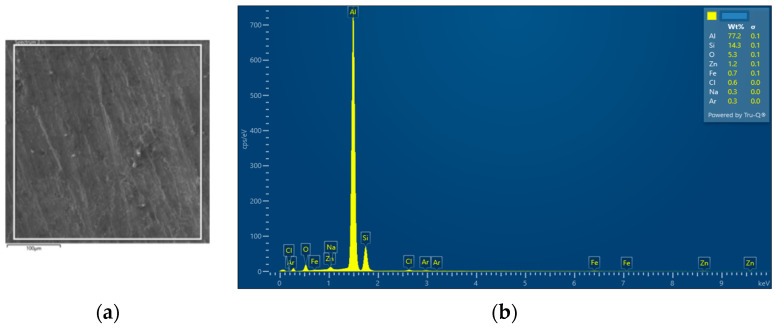
EDX analysis and its evaluation with respect to the local chemical composition: (**a**) chemical composition of the AlSi12 alloy used; (**b**) sample SAM6.

**Figure 19:**
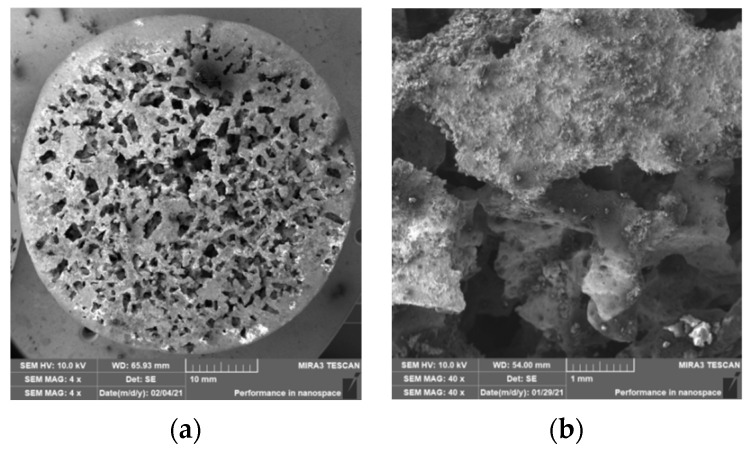
View of the structure of the porous material of AlSi12 alloy: (**a**) detail of the material; (**b**) scanning electron microscope image (Vega 3 Tescan, SEM HV 20.0 kV) of sample SAM7.

**Figure 20:**
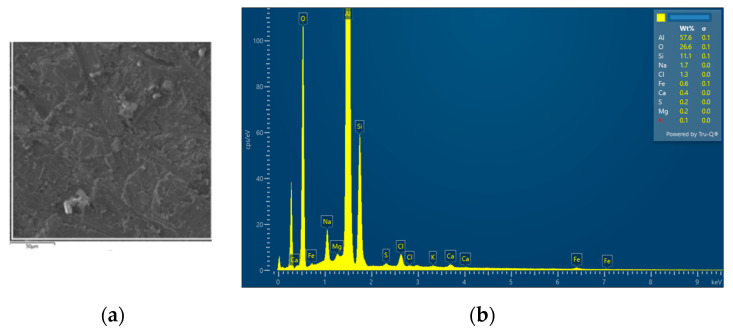
EDX analysis and its evaluation with respect to the local chemical composition: (**a**) chemical composition of the AlSi12 alloy used; (**b**) sample SAM7.

The interconnection of open cells in the structure of aluminium alloy AlSi12 was monitored via a scanning electron microscope (SEM) TESCAN (model Vega 3, company TESCAN, Brno, Czech Republic, HV 20.0 kV). Furthermore, EDX analysis was also performed and the chemical composition of the AlSi12 aluminium alloy in the selected sample location was evaluated. The resulting structures from the electron microscope of each sample (excluding sample SAM2, which was used for the compression test), as well as the EDX analysis results, are shown in Figure 9, Figure 10, Figure 11, Figure 12, Figure 13, Figure 14, Figure 15, Figure 16, Figure 17, Figure 18, Figure 19 and Figure 20. 

In order to increase the clarity of this study, Table 7 shows the results of the local chemical composition of the AlSi12 alloy according to EDX analysis.

An analytical method referred to as energy dispersive spectroscopy (EDX) was applied to analyse the chemical (elemental) composition of the AlSi12 alloy. This analytical approach was based on the detection of characteristic X-rays emitted by sample atoms excited by the impact of an electron beam with kinetic energies ranging from units to hundreds of keV. The characteristic result was the intensity of EDX spectra (cps/eV) of individual elements from a small area of the monitored alloy. As shown in Figure 10, Figure 18 and Figure 20, these spectra showed the presence of a number of accompanying elements (e.g., O_2_; Ca; Fe; S, Mg and Ar). The presence of these elements was caused by the metallurgical preparation of the AlSi12 alloy melt. Some spectra indicated the presence of a higher amount of oxygen varying between 5.3 wt% and 26.6 wt% (see Figure 12, Figure 14, Figure 16, Figure 18 and Figure 20). The elements Na and Cl were also detectable in small amounts; these were mostly residues of the sodium chloride used to produce this porous material. The amount of sodium was from 0.3 wt% to 2.8 wt% and the amount of chlorine was from 0.6 to 3.4 wt%, as demonstrated in Figure 10, Figure 12, Figure 14, Figure 16, Figure 18 and Figure 20. Furthermore, the amount of aluminium and silicon was monitored at locally distributed points in the produced porous samples. This detected amount of aluminum varied from 34.9 wt% to 78.0 wt% and the amount of silicon was from 8.8 wt% to 14.3 wt%. 

**Figure 21:**
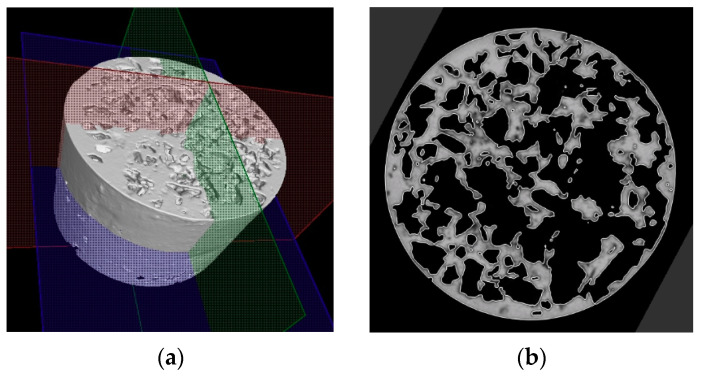

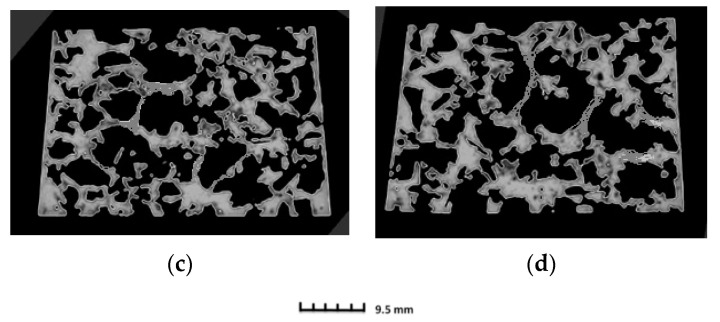
Demonstration of porosity in selected planes of aluminium porous material, AlSi12 sample SAM3, evaluated on a METROTOM 1500 CT ZEISS industrial computed tomograph: diagram of planes of section through porous sample (**a**); cut plane—purple colour (**b**); cut plane—red colour (**c**); cut plane—green colour (**d**).

**Figure 22:**
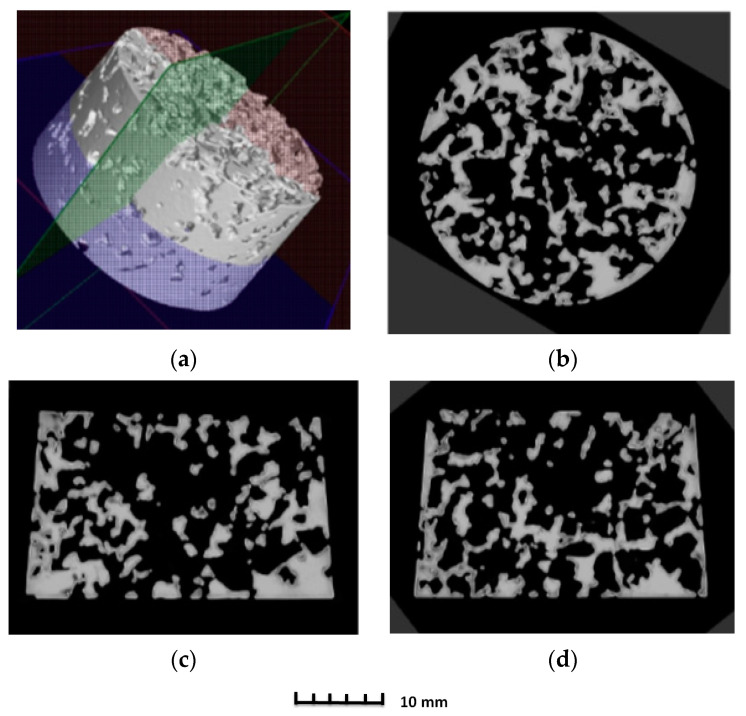
Demonstration of porosity in selected planes of aluminium porous material, AlSi12 sample SAM4, evaluated on a METROTOM 1500 CT ZEISS industrial computed tomograph: diagram of planes of section through porous sample (**a**); cut plane—purple colour (**b**); cut plane—red colour (**c**); cut plane—green colour (**d**).

**Figure 23:**
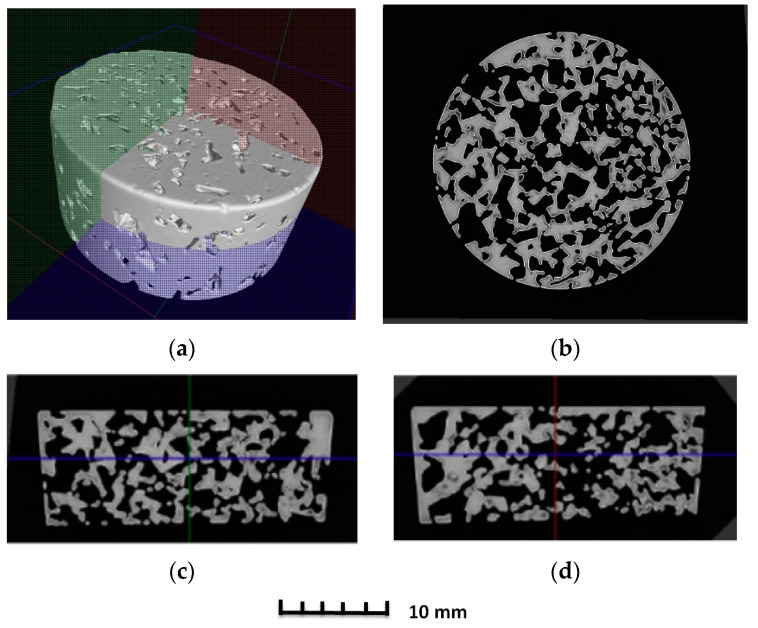
Demonstration of porosity in selected planes of aluminium porous material,.AlSi12 sample SAM6, evaluated on a METROTOM 1500 CT ZEISS industrial computed tomograph: diagram of planes of section through porous sample (**a**); cut plane—purple colour (**b**); cut plane—red colour (**c**); cut plane—green colour (**d**).

The porosity of aluminium samples can be seen in Figure 9, Figure 11, Figure 13, Figure 15, Figure 17 and Figure 19. This porosity was well related to the particle size of the sodium chloride used for production. Simultaneously, the connection of individual cells is evident in the above figures. The more complex nature of the porosity of the selected samples could be observed if the METROTOM 1500 CT ZEISS industrial computed tomograph (model, 1500 company ZEISS, Oberkochen, Germany) was used. These complex structures are shown in Figure 21, Figure 22 and Figure 23.

The same device was used to investigate selected samples (SAM3, SAM4 and SAM6) of porous aluminium materials made from AlSi12 alloy. Figure 21, Figure 22 and Figure 23 show the final 3D scan with the positions of the relevant planes as well as the individual sections along these planes.

## 4. Discussion

The initial results regarding the production of porous materials by pressing sodium chloride particles into molten aluminium alloy indicated that this idea represents a genuinely feasible production technology. In fact, porous aluminium materials are mostly produced by infiltrating the melt between the particles of sodium chloride or other suitable materials. However, this melt infiltration method is based on the creation of a vacuum in the “foundry” mould. Consequently, a specially designed melting furnace with an opening in the upper part must be used. Moreover, a foundry mould, which is designed for the inlet of argon and for connecting the mould to a vacuum pump, is also needed. The pressing method is associated with a strict metallurgical preparation of the relevant aluminium alloy melt. At the same time, a precise methodology for the production process must be developed, including the setting of press working parameters. In addition, it is also important to ensure a uniform distribution of sodium chloride particles throughout the volume of metal materials and the thermal preparation of the sodium chloride itself. A similar method for the production porous materials (lead alloys with antimony) using sodium chloride was carried out by the authors of [20]. Results concerning the production of samples of porous aluminium materials with the same shape (from aluminium alloy AlSi12 and four sizes of sodium chloride particles: 1 to 3 mm, 3 to 5 mm, 5 to 7 mm and 8 to 10 mm) confirmed that the proposed and verified methodology is suitable for production. At the same time, it can be stated that the obtained samples are compact in shape with open cells, which are related to the size of the sodium chloride particles used. Using a scanning electron microscope, it was detected that the open cells are interconnected on the sample SAM4 surfaces (see, e.g., Figure 13). A much stricter method, in light of the evaluation of the internal structure, was carried out using a Zeiss METROTOM 1500 for industrial computed tomography. The porosity of the selected samples was observed along the different planes, as shown in Figure 21, Figure 22 and Figure 23 (samples SAM3, SAM4 and SAM6). It is evident that the outline of the samples was solid and that the metallic material was never missing. The realization of the target of producing porous materials with a regular distribution of open cells is evident from the analysed samples. Furthermore, Figure 21, Figure 22 and Figure 23, demonstrate that there are areas in which large cells are located in some central parts of the tested samples, which definitely contribute to the heterogeneity of produced materials. It must be noted that the production methodology will need to be corrected in future. Attention must be taken to ensure a uniform distribution of salt in the volume of the produced materials. Furthermore, the preparation process, which involves the melt along with the required amount of sodium chloride and their subsequent compression by a mechanical press in a metal foundry mould, must be conducted precisely. Such observation of the regular open-cell distribution in porous metallic material was also undertaken by the authors of [16], and it was concluded that with the use of higher sodium chloride contents in the production of porous materials, the heterogeneity of the open-cell distribution also increases (higher amounts of sodium chloride particles combine). As shown in our results related to the observation of the internal structure, the smaller sodium chloride particles are, in this case, preferable to ensure a regular open-cell distribution. Porous materials that do not have a regular arrangement of cells are suitable, e.g., for absorption applications or can be used for filtration processes. By evaluating the density of prepared samples of porous aluminium materials that have almost the same dimensions, it was found that the density is connected to the size of the sodium chloride particles used. The presence of larger sodium chloride particles led to a larger volume of cells in the porous metallic material. Conversely, smaller particles of sodium chloride formed a smaller cell volume. Therefore, it could be expected that the produced porous material with larger sodium chloride particles would have the lowest density, and that samples of porous material with smaller particles would contribute to the higher density of the given porous materials. This hypothesis was also confirmed by the calculated density values of produced samples. More precisely, using sodium chloride particles of 8 to 10 mm size, the density of the samples was approximately 980 kg·m^−3^. In the case of sodium chloride particles with an average size of 6 mm, the average density of samples was approx. 1000 kg·m^−3^. Using sodium chloride particles with an average size of 4 mm, the average density of the samples reached a value of 1090 kg·m^−3^. Finally, in the case of sodium chloride particles with an average size of 2 mm, the average density of samples was about 1186 kg·m^−3^.

The relatively complex production of samples of porous materials was based on the theoretical assumption that 50% of the volume would be occupied by sodium chloride and 50% of the volume would be occupied by AlSi12. To ensure the technical performance of these experiments, the respective volumes were converted to the weight of both types of components (NaCl and AlSi12 alloys). In particular, the homogeneity of NaCl was closely related to the particle size of NaCl. Moreover, we were not able to provide exactly the same volume of melt. For this reason, the individual samples had different heights. The aforementioned circumstances also affected the resulting density of the produced samples of porous materials. 

In further research, it will be necessary to focus on ensuring the regular stratification of NaCl particles and to specify the methodology used for measuring the amount of aluminium alloy melt.

## 5. Conclusions

The methodology used for the production of porous aluminium alloy material, with the use of sodium chloride particles, led to the following conclusions being reached:

(a) For the implementation of the experiments, a hydraulic forming press is required to develop a minimum pressure in the range of 100 MPa to 150 MPa. At the same time, it is necessary to prepare a foundry mould with a cavity that has a simple shape.
(b) Moreover, considerable attention must be paid to the metallurgical preparation of the melt, as well as its treatment and temperature measurement, before its casting into the mould cavity. It is also necessary to preheat the mould to 550 °C and to preheat the sodium chloride to 150 °C. In addition, treatment of the mould cavity with a suitable protective coating is required.
(c) For the production of porous materials, a 50% volume of sodium chloride and a 50% volume of aluminium alloy AlSi12 were used. Four different sizes of sodium chloride particles were used: from 8 to 10 mm, from 5 to 7 mm, from 3 to 5 mm and from 1 to 3 mm, respectively. Through the calculation of the material density, it can be found that larger salt particle sizes led to the lower density of produced sample. This fact is consistent with the well-known general theory of porous materials. The density of the produced porous samples varied within the range of 975 to 1186 kg·m^−3^. Note that the same material samples, without any porosity, had a density of 2650 kg·m^−3^. In sum, the porous material samples had a density that was from 2.0 to 2.7 times smaller than raw material without porosity, and the calculated porosity of the produced porous samples varied from 55% to 62% (the larger the sodium chloride particles, the greater the porosity).

## Figures and Tables

**Table 1:** Chemical composition of NaCl.

Sample NaCl	Chemical Composition NaCl of wt%						
	Cl	Na	Ca	S	Si	K	Br
I.	58.8	40.7	0.17	0.13	0.08	0.04	0.08
II.	58.9	40.6	0.21	0.14	-	-	0.15

Note: The device XRF Bruker S8 Tiger works with an accuracy of ±3 (wt%).

**Table 2:** Chemical composition of the AlSi12 aluminium alloy used.

Chemical Composition of Aluminium Alloy AlSi12 (wt%)										
Si	Fe	Mn	Cu	Zn	Ti	V	Mg	Na	Pb	Al
According to EN AC 44300										
10.5–13.5	0.45–0.90	0.55	0.08	0.15	0.15	-	-	-	-	Balance
Real chemical composition of used alloy										
12.2	0.44	0.45	0.04	0.12	0.1	0.006	0.001	0.005	0.004	Balance

Note: The spectrometer works with an accuracy of ±5 (wt%).

**Table 3:** Calculated values weight of AlSi12 alloy and sodium chloride.

**Calculus of AlSi12 Aluminum Alloy Weight**		
Weight AlSi12 m_AlSi12_ (kg)	Volume AlSi12 VAlSi12 (m^3^)	Density AlSi12 ρAlSi12 (kg·m^−3^)
0.079	2.987 × 10^−5^	2650
**Calculus of Sodium Chloride Weight**		
Weight NaCl m_NaCl_ (kg)	Volume NaCl V_NaCl_ (m^3^)	Density NaCl ρ_NaCl_ (kg⋅m^−3^)
0.065	2.987 × 10^−5^	2165

**Table 4:** Basic dimensions and density of produced samples from aluminum porous material.

No. of Sample	Size of NaCl Particles	Basic Dimensions			Values for Density Computation			
		ØD	Ød	Height	Weight	Volume	Density	Relative Density
				h	m	V	ρ	ρ_REL_
	(mm)	(mm)	(mm)	(mm)	(kg)	(m^3^)	(kg·m^−3^)	(1)
SAM1	8 to 10	45.9	40.1	27.5	0.0390	3.997 × 10^−5^	975	0.37
SAM2	8 to 10	45.5	41.0	28.3	0.0411	4.159 × 10^−5^	988	0.37
SAM3	5 to 7	45.3	42.2	24.6	0.0367	3.678 × 10^−5^	997	0.38
SAM4	5 to 7	46.5	42.3	25.0	0.0389	3.870 × 10^−5^	1005	0.38
SAM5	3 to 5	46.2	44.1	20.5	0.0359	3.281 × 10^−5^	1094	0.41
SAM6	3 to 5	46.5	44.4	16.2	0.0285	2.610 × 10^−5^	1085	0.41
SAM7	1 to 3	46.3	42.7	22.5	0.0415	3.500 × 10^−5^	1186	0.45

Comments: uncertainty of length scales are ± 0.05 mm, weight is ±0.01 g, volume is approx. ±10^−7^ m^3^ and density is 7%.

**Table 5:** Basic properties of base material and porous aluminium alloys (NaCl squeezed into aluminium alloy melt)—samples SAM1–SAM4.

No. of Sample		SAM1	SAM2	SAM3	SAM4
Size of NaCl Particles	(mm)	8 to 10	8 to 10	5 to 7	5 to 7
Density of AlSi12 (base material)	*ρ_B.M.(AlSi12)_*	2650	2650	2650	2650
	(kg·m^−3^)				
Volume of AlSi12 (base material)	*V_B.M.(AlSi12)_*	3.992 × 10^−5^	4.156 × 10^−5^	3.696 × 10^−5^	3.869 × 10^−5^
	(m^3^)				
Weight of AlSi12 (base material)	*m_B.M.(AlSi12)_*	0.106	0.110	0.098	0.103
	(kg)				
Density of porous AlSi12	*ρ_P.(AlSi12)_*	975	988	997	1005
	(kg·m^−3^)				
Relative density of AlSi12 (porous/base)	*ρ_REL.(AlSi12)_*	0.37	0.37	0.38	0.38
	(kg·m^−3^)				
Volume of porous AlSi12	*V_P.(AlSi12)_*	4.012 × 10^−5^	4.159 × 10^−5^	3.678 × 10^−5^	3.870 × 10^−5^
	(m^3^)				
Weight of porous AlSi12	*m_P.(AlSi12)_*	0.039	0.041	0.037	0.039
	(kg)				
Porosity of porous AlSi12	*p*	63	63	62	62
	(%)				
	MPa				

**Table 6:** Basic properties of base material and porous aluminium alloys (NaCl squeezed into aluminium alloy melt)—samples SAM5–SAM7.

No. of Sample		SAM5	SAM6	SAM7
Size of NaCl particles	(mm)	3 to 5	3 to 5	1 to 3
Density of AlSi12 (base material)	*ρ* *_B.M.(AlSi12)_*	2650	2650	2650
	(kg·m^−3^)			
Volume of AlSi12 (base material)	*V_B.M.(AlSi12)_*	3.281 × 10^−5^	2.627 × 10^−5^	3.500 × 10^−5^
	(m^3^)			
Weight of AlSi12 (base material)	*m_B.M.(AlSi12)_*	0.087	0.068	0.093
	(kg)			
Density of porous AlSi12	*ρ* *_P.(AlSi12)_*	1094	1085	1186
	(kg·m^−3^)			
Relative density of AlSi12 (porous/base)	*ρ* *_REL.(AlSi12)_*	0.41	0.41	0.45
	(kg·m^−3^)			
Volume of porous AlSi12	*V_P.(AlSi12)_*	3.281 × 10^−5^	2.627 × 10^−5^	3.500 × 10^−5^
	(m^3^)			
Weight of porous AlSi12	*m_P.(AlSi12)_*	0.036	0.029	0.042
	(kg)			
Porosity of porous AlSi12	*p*	59	59	55
	(%)			

**Table 7:** Local chemical composition of AlSi12 alloy according to EDX analysis.

Chemical Composition of wt% (EDX Analysis)													
Sample	Al	Si	Cl	Na	Zn	Fe	O	Ca	Mg	S	Ar	B	P
SAM1	34.9	8.8	3.0	2.8	0.8	0.5	48.2	0.5	0.1	0.3	-	-	0.1
SAM2	the sample was not tested												
SAM3	76.3	10.1	1.1	1.3	-	-	11.3	-	-	-	-	-	-
SAM4	76.8	10.0	3.4	1.6	-	1.1	7.1	-	-	-	-	-	-
SAM5	78.0	14.2	0.8	0.5	-	-	6.4	-	-	-	-	-	-
SAM6	77.2	14.3	0.6	0.3	1.2	0.7	5.3	-	-	-	0.3	-	-
SAM7	57.6	11.1	1.3	1.7	-	0.6	26.6	0.4	0.2	0.3	-	0.1	-

## Data Availability

All data are available from the authors.

## Appendix A

The uncertainty of the volume is given by the following formula, which is derived from the Equation (A1).

(A1) $$Var_{V}\sqrt{{\left(\frac{\partial V}{\partial H}Var_{H}\right)}^{2}+{\left(\frac{\partial V}{\partial d_{1}}Var_{D,d}\right)}^{2}+{\left(\frac{\partial V}{\partial d_{2}}Var_{D,d}\right)}^{2}}$$

where *Var_H_* is 0.05 and *Var_D,d_* is 0.05 as well.

The partial ratios are defined below:

(A2) $$\frac{\partial V}{\partial H}=\frac{1}{12}\pi \left(d^{2}+Dd+D^{2}\right)\;\frac{\partial V}{\partial D}=\frac{1}{12}\pi \left(d+2D\right)\;\frac{\partial V}{\partial d}=\frac{1}{12}\pi \left(2d+D\right)$$
